# In vitro pro-inflammatory enzyme inhibition and anti-oxidant potential of selected Sri Lankan medicinal plants

**DOI:** 10.1186/s12906-018-2335-1

**Published:** 2018-10-03

**Authors:** Hettiarachchige Dona Sachindra Melshandi Perera, Jayanetti Koralalage Ramani Radhika Samarasekera, Shiroma Mangalika Handunnetti, Ovitigala Vithanage Don Sisira Jagathpriya Weerasena, Hasitha Dhananjaya Weeratunga, Almas Jabeen, Muhammad Iqbal Choudhary

**Affiliations:** 1 Industrial Technology Institute (ITI), 363, Bauddhaloka Mawatha, Colombo, 07 Sri Lanka; 2 Institute of Biochemistry, Molecular Biology and Biotechnology, University of Colombo, 90, Cumaratunga Munidasa Mawatha, Colombo, 03 Sri Lanka; 3 Dr. Panjwani Center for Molecular Medicine and Drug Research, International Center for Chemical and Biological Sciences, University of Karachi, Karachi, 75270 Pakistan; 4 H. E. J. Research Institute of Chemistry, International Center for Chemical and Biological Sciences, University of Karachi, Karachi, 75270 Pakistan

**Keywords:** Anti-inflammatory, Enzyme inhibition, Anti-oxidant, Medicinal plants, *F. indica*, Gas chromatography-mass spectrometry, High performance liquid chromatography

## Abstract

**Background:**

The extracts of the ten selected Sri Lankan medicinal plants have been traditionally used in the treatment of inflammatory mediated diseases. The extracts were investigated for anti-inflammatory and anti-oxidant potential in vitro to identify bio-active extracts for further chemical characterization.

**Methods:**

In vitro anti-inflammatory activities of total ethanol extracts were investigated measuring the inhibitory activities of four pro-inflammatory enzymes, arachidonate-5- lipoxygenase (A5-LOX), hyaluronidase (HYL), xanthine oxidase (XO) and inducible nitric oxide (iNO) synthase. Cytotoxicity of extracts were determined by MTT assay. Oxidative burst inhibition (OBI) on human whole blood (WB) and isolated polymorphoneutrophils (PMNs) was carried out for a selected bio-active extract. Anti- oxidant activities of the extracts were determined by 2,2-diphenyl-1-picrylhydrazyl (DPPH) free radical scavenging, ferric reducing antioxidant power (FRAP), ferrous ion chelation (FIC) and oxygen radical absorbance capacity (ORAC) assays. Total polyphenol and total Flavonoid contents of the extracts were also determined. The most active plant extract was analysed using Gas chromatography-Mass spectrometry (GC-MS) and High Performance Liquid Chromatography (HPLC).

**Results:**

The ethanol bark extract of *Flacourtia indica* showed the highest A5-LOX (IC_50_: 22.75 ± 1.94 g/mL), XO (70.46 ± 0.18%; 250 μg/mL) and iNOs inhibitory activities on LPS- activated raw 264.7 macrophage cells (38.07 ± 0.93%; 500 μg/mL) with promising OBI both on WB (IC_50_: 47.64 2.32 μg/mL) and PMNs (IC_50_: 5.02 0.38 μg/mL). The highest HYL inhibitory activity was showed by the leaf extracts of *Barathranthus nodiflorus* (42.31 ± 2.00%; 500 μg/mL) and *Diospyros ebenum* (41.60 ± 1.18%; 500 μg/mL). The bark and leaf extracts of *Callophyllum innophyllum* (IC_50_: 6.99 ± 0.02 μg/mL) and *Symplocus cochinchinesis* (IC_50_: 9.85 ± 0.28 μg/mL) showed promising DPPH free radical scavenging activities. The GC-MS analysis of ethanol bark extract of *F. indica* showed the presence of two major bio-active compounds linoleic acid ethyl ester and hexadecanoic acid, ethyl ester (> 2% peak area). The HPLC analysis showed the presence polyphenolic compounds.

**Conclusion:**

The ethanol bark extract of *F. indica* can be identified as a potential candidate for the development of anti-inflammatory agents, which deserves further investigations. The bio-active plant extracts may be effectively used in the applications of cosmetic and health care industry.

## Background

In Sri Lanka, medicinal plants have always been used and still remain a major source in the treatment of number of diseases including inflammatory and oxidative-stress associated chronic diseases. Free radicals can be either beneficial or deleterious to the body depending on the level. The excess levels of free radicals will cause damage to most cellular macromolecules such as proteins (enzymes), lipids and DNA leading to a condition called oxidative stress [1]. Oxidative stress has been recognized as a key factor in the pathogenesis of many diseases including inflammatory diseases [2]. The excess of reactive oxygen species (ROS) generated will lead to inflammation by stimulating cytokines and activation of pro-inflammatory enzymes such as lipoxygenase, hyaluronidase, inducible nitric oxide synthase and xanthine oxidase [3]. Lipoxygenases are capable of generating lipid mediators such as leukotrines and prostaglandins, which can provoke several inflammatory diseases such as bronchial asthma, allergic rhinitis, cardiovascular diseases, rheumatoid arthritis and certain types of cancer [4]. Hyaluronidase will lead to degranulation of mast cells and release inflammatory mediators leading to several pathological conditions including rheumatoid arthritis [5]. Upon activation of inflammatory cells, inducible nitric oxide synthase (iNOs) will generate excessive amount of nitric oxide (NO), which can cause inflammation [6]. Xanthine oxidases also play a major role in the metabolic disease called gout, which is closely associated with inflammation and some other inflammatory mediated diseases due to the formation of free radicals during the catalytic function of the enzyme. It is evident that these pro-inflammatory enzymes play an important role in the pathogenesis of inflammation via different pathways. Hence, inhibition of these enzymes is considered as targets for the management of diseases associated with oxidative stress and inflammation [7].

Oxidative burst is characterized by the production and rapid release of reactive oxygen species (ROS) from immune cells, mainly by neutrophils. Though it is considered to play an important role as a defense mechanism in phagocytosis, the higher levels of ROS released during the oxidative burst has been identified to cause severe tissue injury and inflammation. Therefore inhibition of oxidative burst has been recognized as an interesting strategy in the research arena of anti-inflammatory drug research [8]. Anti-oxidants also play an important role in the management of inflammation. The efficacy of antioxidants and anti-inflammatory drugs derived from medicinal plants in the management of inflammatory diseases has been extensively documented. In this concern, medicinal plants are considered as valuable sources of potential therapeutic agents. A number of modern drugs have been isolated from medicinal plants based on the traditional use. There is an emerging interest in the use of natural products mainly those derived from medicinal plants in therapeutic applications [9]. In Sri Lanka, the practice of Ayurveda and traditional system of medicine has been implemented systematically for more than two thousand years to treat various diseases including inflammatory mediated diseases. Around 1414 of plant species including several endemic species have been used for the treatment and prevention of diseases. Among them, around 200 species are in general use and of them, nearly 50 species have been identified as heavily used plant species in Ayurveda and traditional system of medicine. With the estimated annual consumption of 2.2 million Kg, the potential for commercial exploitation of medicinal plants has risen high [10]. In the existing scenario of emerging global interest for natural products of high therapeutic potential, exploring bio-activities of Sri Lankan medicinal plants is of great importance and high demand to support traditional claims as well as to discover unexploited bio-active properties. Moreover, the bio-assay guided isolation of bio-active compounds from identified bio-active medicinal plant extracts may come up with more effective and safer therapeutic agents against various diseases including inflammatory diseases and other oxidative stress associated chronic diseases. Also the bioactive ingredients are of high commercial potential in health care and pharmaceutical industries.

Based on this rationale, we investigated A5-LOX, hyaluronidase, xanthine oxidase, nitric oxide production and oxidative burst inhibitory properties along with anti-oxidant capacities of ten selected Sri Lankan medicinal plants, which have been used in the traditional system of medicine in the management of diseases, associated with inflammation (Table 1).

**Table 1:** Traditional uses of ten Sri Lankan medicinal plants

Plant name/(FAMILY)	Local name/English name	Part used in the study/Voucher specimen No.	Traditional uses
*Sphaeranthus indicus* L. (Asteraceae)	Mudumahana/East India Globe Thistle	Leaf/SEL/15/11	Swelling in the neck, acute laryngitis and bronchitis, piles [42].
*Acronychiapedunculata* L. (Rutaceae)	Ankenda/Claw flowered laurel.	Leaf/APL/15/15	Skin diseases, rheumatism, ulcers asthma [43].
*Calophyllum innophyllum* Linn.(Clusiaceae)	Domba/Alexandrian laurel	Bark/CIB/15/21	Skin diseases, piles, sore eyes, migraine [44].
*Symplocos cochinchinesis* (Lour.) S. Moore. (Symplocaceae)	Sewalabombu/Lodh tree	Bark/SCB/15/27	Leprosy, tumors, menorrhagia, inflammation and uterine problems [45].
*Tinospora cordifolia* (Willd.) (Menispermaceae)	Rasakinda/heartleaf moonseed	Bark/TCB/15/32	Skin diseases, Jaundice, Diabetes, rheumatic pain, syphilis [46].
*Flacourtia indica* (Burm.f. Merr.) (Flacourica)	Uguressa/Governor’s Plum	Bark/FIB/15/34	Rheumatoid arthritis, gout, intermittent fever [40, 47].
*Leucus zeylanica* L. (Lamiaceae)	Gata thumba/Ceylon slitwort	Leaf/LZL/15/41	Jaundice, scorpion, snake bite [48].
*Barathranthus nodiflorus* Thw. (Loranthaceae)	Pilila	Leaf/BNL/15/44	Bone fractures [49].
*Diospyros ebenum* J.Koenig ex Retz (Ebenaceae)	Kaluwara/Ceylon ebony	Leaf/DEL/15/47	Snake bite, diarrhoea, ulcers, biliousness [32].
*Argyreia populifolia* Choisy (*C*onvolvulaceae)	Elephant creeper	Leaf/APL/15/48	Swellings [50].

## Methods

### Chemicals and equipment

A5-LOX (soybean), linoleic acid, baicalein, hyaluronic acid potassium salt (human umbilical cord), hyaluronidase (bovine testes), calcium chloride, sodium hydroxide, p-Dimethylaminobenzaldehyde (PDMAB), sodium borate, tannic acid, xanthine oxidase (bovine milk), xanthine, allopurinol, Dulbecco’s modified Eagle’s medium (DMEM), fetal calf serum (FCS), bacterial lipopolysaccharide (LPS), trypsin, 3-(4,5-dimethylthiazol-2-yl)-2,5-diphenyltetrazoliumbromide (MTT), NG-Monomethyl-L-argininep-dimethylamino benzaldehyde (NMMA), HBSS^++^ (Hanks Balanced Salt Solution, containing calcium chloride and magnesium chloride) [Sigma, St. Louis, USA], serum opsonized zymosan (SOZ) [Fluka, Buchs, Switzerland], HBSS^—^ (Hanks Balanced Salt Solution without calcium chloride and magnesium chloride),folin-ciocalteu reagent, gallic acid, quercetin, ethylenediaminetetraacetic acid disodium salt (EDTA-Na_2_), dimethylsulfoxide (DMSO), 2,2-diphenyl-2-picryl-hydrazyl (DPPH), 6-hydroxy-2-5-7-8-tetramethylchroman-2-carboxylic acid (trolox), potassium persulphate,2,2-azobis (2-amidinopropane) dihydrochloride (AAPH), sodium fluorescein, 2,4,6-tripyridyl-s-triazine (TPTZ) and 4,4′-disulfonic acid sodium salt (ferrozine) were purchased from Sigma-Aldrich (USA). All chemicals and reagents used in the experiment were of analytical grade. The bio-assays were performed using high throughput micro-plate readers (Spectra Max Plus384, Molecular Devices, USA and Spectra Max-Gemini EM, Molecular Devices Inc., USA).

### Plant material collection and preparation of extracts

Fresh plant materials, leaves of *Sphaeranthus indicus* L*.*(Asteraceae)*,* leaves of *Acronychia pedunculata* L (Rutaceae)*,* bark of *Calophyllum innophyllum* Linn. (Clusiaceae)*,* bark of *Symplocos cochinchinesis* (Lour.) S. Moore*.* (Symplocaceae)*,* bark of *Tinospora cordifolia* (Willd.) (Menispermaceae)*,* bark of *Flacourtia indica* (Burm.f. Merr.) (Salicaceae), leaves of *Leucus zeylanica* L. (Lamiaceae)*,* leaves of *Barathranthus nodiflorus* Thw. (Loranthaceae), leaves of *Diospyros ebenum* J.Koenig ex Retz (Ebenaceae) and leaves of *Argyreia populifolia* Choisy (*C*onvolvulaceae) were collected from Gampaha, Sri Lanka.

Plants were identified by Ms. S. Sugathadasa and voucher specimens were deposited at Herbal Technology Section, Industrial Technology Institute, Sri Lanka (leaves of *Sphaeranthus indicus* L: SEL/15/11*,* leaves of *Acronychia pedunculata* L: APL/15/15*,* bark of *Calophyllum innophyllum* Linn.: CIL/15/21*,* bark of *Symplocos cochinchinesis* (Lour.) S. Moore.: SCB/15/27*,* bark of *Tinospora cordifolia* (Willd): TCB/15/32*,* bark of *Flacourtia indica* (Burm.f. Merr.): FIB/15/34, leaves of *Leucus zeylanica* L.: LZL/15/41*,* leaves of *Barathranthus nodiflorus* Thw.: BNL/15/44, leaves of *Diospyros ebenum* J.Koenig ex Retz: DEL/15/47 and leaves of *Argyreia populifolia* Choisy: APL/15/48) Plant materials were shade-dried under well-ventilated conditions (Relative humidity: 65–75%), at room temperature (25 ± 2 °C) for 72 h and ground to make coarse powder using a mechanical grinder [11, 12]. Powdered plant materials (100 g) were soaked in ethanol (500 mL) overnight and stirred for 1 h using a mechanical stirrer at room temperature (25 ± 2 °C) followed by suction filtration through a celite bed, packed in a sintered funnel. The filtrates were concentrated under reduced pressure at 40 °C using a rotary evaporator to obtain the ethanol extracts [11]. The solvent free extracts were stored in air-tight glass containers at − 20 °C until used [13].

### Enzyme inhibitory activity

#### Arachidonate 5-lipoxygenase (A5-LOX) inhibitory assay

A5-LOX inhibitory activity of plant extracts was determined by a modified spectrometric method [14]. Plant extracts were assayed within the concentration range of 10–1000 μg/mL. Briefly, sodium phosphate buffer (110 μL,100 mM, pH 8.0), plant extracts dissolved in methanol (10 μL), and A5-LOX solution (55 μL) were incubated for 10 min at 25 °C followed by the addition of linoleic acid solution (25 μL, 0.08 mM). Absorbance was measured at λ = 234 nm for 10 min at 25 °C. Percentage inhibition of A5-LOX was determined by comparison of reaction rates of extracts relative to control using the formula (*E* − *S*)/*E* × 100, where *E* and *S* are activities of the enzyme with and without extracts, respectively. IC_50_ values were determined. Baicalein was used as the reference standard.

#### Hyaluronidase inhibitory assay

Hyaluronidase inhibitory activity of plant extracts was evaluated by a spectrometric method with modifications [15]. Extracts were assayed at the concentrations of 100 and 500 μg/mL. Extracts (50 μL) were incubated with hyaluronidase enzyme solution (10 μL) at 37 °C for 10 min followed by the addition of calcium chloride (12.5 mM, 20 μL) and re-incubation at 37 °C for 10 min. Sodium hyaluronate (50 μL) was added to the reaction mixture and incubated at 37 °C for 40 min followed by the addition of Sodium hydroxide (0.9 M, 10 μL) and Sodium borate (0.2 M, 20 μL) and incubation at 100 °C for 3 min. p-Dimethylaminobenzaldehyde (PDMAB), (50 μL, 67 mM) was added to the reaction mixture and incubated at 37 °C for 10 min. Absorbance was measured at λ = 585 nm. Percent enzyme inhibition was calculated as given below, compared to the control. Tannic acid was used as the reference standard.

Inhibition (%) = [(Abs. control – Abs. sample)/Abs. control] × 100.

#### Xanthine oxidase inhibitory activity

Xanthine oxidase inhibitory activity of plant extracts was determined by a kinetic method [16] with slight modifications. Extracts were tested at the assay concentration of 250 μg/mL. Briefly, sodium phosphate buffer (150 μL, 50 mM, pH 7.4), extracts (10 μL) and xanthine oxidase solution (10 μL) were incubated at 25 °C for 10 min. The reaction was then initiated with the addition of xanthine solution (0.1 mM). Absorbance was monitored with the change of absorbance at λ = 295 nm for 15 min at 25 °C. Percentage inhibition of xanthine oxidase was calculated using the formula (*E* − *S*)/*E* × 100, where *E* is the activity of enzyme without extracts and *S* is the activity of enzyme with extracts. Allopurinol was used as the reference standard.

#### Nitric oxide production inhibitory activity and viability of LPS-activated RAW 264.7 macrophages

##### Cell culture

Murine macrophage (RAW 264.7) cell lines were purchased from ATCC, VA, USA. The RAW 264.7 cells were cultured and maintained in DMEM, supplemented with streptomycin sulfate (100 μg/mL), penicillin G sodium (100 units/mL), amphotericin B (0.25 μg/mL) and 10% fetal bovine serum (FBS) (Humidified atmosphere, 5% CO_2_, 37 °C). Cells were split twice a week.

Monolayer cells were plated in 96-well micro-plates at a density of 1 × 10^5^ cells/well followed by the incubation (humidified atmosphere, 5% CO_2_, 37 °C) for 24 h. The plated cells were treated with extracts (500 μg/mL) and incubated for 30 min (humidified atmosphere, 5% CO_2_, 37 °C), followed by the incubation with bacterial lipopolysaccharide (LPS, 1 μg/mL) for 24 h [17].

##### Nitric oxide production inhibition

The inhibition of nitric oxide production was determined using the Griess assay [6]. After 24 h incubation with LPS, cell culture supernatants (100 μL) were reacted with Griess reagent (100 μL) and incubated for 10 min at room temperature and absorbance was measured at λ = 540 nm. The nitrite concentration was determined using a standard curve of sodium nitrite (y = 0.012× + 0.036, R^2^ = 0.999). Percentage inhibition of nitric oxide formation by extracts was calculated [18].

##### Cell viability

The cytotoxicity of the extracts on RAW 264.7 cells was determined by MTT assay [19]. Cells were initially incubated (humidified atmosphere, 5% CO_2_, 37 °C) for 6 h and with plant extracts (500 μg/mL) for 30 min. The cells were treated with LPS (1 μg/mL) and incubated for 24 h. MTT solution (20 μL, 5 mg/mL in PBS) and FBS free DMEM (180 μL) were added to the cells and incubated (humidified atmosphere with 5% CO_2_ at 37 °C) for 4 h. DMSO (100 μL) was added to dissolve the formed formazan salt and absorbance was measured at λ = 570 nm. Percentage cell viability was determined [18].

##### Oxidative burst inhibition

Oxidative burst inhibition assay was conducted at Dr. Panjwani Center for Molecular Medicine and Drug Research, International Centre for Chemical and Biological Sciences, University of Karachi, Pakistan. The institute has obtained the ethical clearance for studies on human blood from independent ethics committee, ICCBS, UoK. No: ICCBS/IEC-008-BC-2015/Protocol/1.0.

##### Isolation of human polymorphoneutrophils (PMNs)

Venous blood was collected from a healthy adult male volunteer (25–30 years age) to a heparinized tube and density gradient centrifugation was carried out to isolate neutrophils [20]. Briefly, whole blood (10 mL), HBSS^—^ (10 mL) and lympho separation medium (LSM, 10 mL) were mixed and kept at room temperature for 45 min for serum separation. The separated serum was centrifuged at 400 g for 20 min and sedimented cells were re-centrifuged with an equal volume of LSM at 300 g, 4 °C for 10 min. The cells were re-suspended in HBSS^—^ and cell count was adjusted to 1 x 10 ^6^ cells/ mL.

##### Chemiluminescence assay

Luminol-enhanced chemiluminescence assay was performed according to a kinetic method [21] with modifications. Briefly, 25 μL of diluted whole blood in HBSS^++^ was incubated with the plant extract (25 μL) at 37 °C for 15 min and 25 μL of serum opsonized zymosan (SOZ) and 25 μL of luminol were added into each well, except blank wells. The level of the ROS was recorded and inhibition of ROS production (%) was calculated. IC_50_ values were determined. Ibuprofen was used as the reference standard.

### Antioxidant activity

#### 2,2-diphenyl-2-picryl-hydrazyl (DPPH) free radical scavenging activity

The DPPH free radical scavenging activity of plant extracts was determined using a spectrophotometric method with modifications [22]. Extracts were assayed within the concentration range of 10–500 μg/mL. Extracts (100 μL) were incubated with DPPH solution (40 μg/mL, 200 μL) at room temperature (25 ± 2 °C) in dark for 10 min and absorbance was measured at 517 nm. The DPPH free radical scavenging activity was calculated using the following equation and IC_50_ values were determined. Trolox was used as the reference standard.

Scavenging activity (%) = [(Abs. control – Abs. sample)/Abs. control]   ×   100.

#### Ferric reducing antioxidant power (FRAP)

The assay was performed according to a spectrophotometric method [23] with slight modifications. Extracts were tested within the assay concentration range of 25–500 μg/ mL. Extracts (50 μL) were incubated with freshly prepared FRAP reagent (Acetate buffer of 300 mM and pH 3.6, TPTZ in 10 mM in 40 mM HCl, and 20 mM ferric chloride hexahydrate solution mixed at 10:1:1, *v*/v/v) (150 μL) at room temperature (25 ± 2 °C) for 8 min. Absorbance was recorded at λ = 593 nm. FRAP of extracts was expressed as mg trolox equivalents (TE)/g of extract using a standard curve of trolox (y = 0.008× + 0.046, R^2^ = 0.996).

#### Ferrous ion chelating (FIC) activity

Ferrous ion chelating activity was determined according to a spectrophotometric method [24] with modifications. Plant extracts were assayed in the assay concentration range of 100–5000 μg/mL.^.^ Extracts (140 μL) were incubated with ferrous sulfate solution (1 mM, 20 μL) at room temperature (25 ± 2 °C) for 10 min. After the incubation, ferrozine (40 μL) was added to the reaction mixture and re-incubated at room temperature (25 ± 2 °C) for 10 min. Absorbance was measured at 562 nm. Percentage chelating effect was calculated with compared to control based on the following equation and IC_50_ values were determined. EDTA-Na_2_ was used as the reference standard.

Chelating activity (%) = [(Abs. control – Abs. sample)/Abs. control] × 100.

#### Oxygen radical absorbance capacity (ORAC)

The oxygen radical absorbance capacity (ORAC) assay was conducted using a kinetic method [25] with modifications. Plant extracts were assayed in the assay concentration range of 1–100 μg/mL. Extracts (10 μL) were pre-incubated with phosphate buffer (40 μL) and fluorescein solution (4.8 μM, 100 μL) at 37 °C for 10 min and freshly prepared AAPH solution (40 μg/mL, 50 μL) was added. The decay of fluorescein was monitored at 1 min intervals for 35 min at the wavelengths of 494 nm (excitation) and λ = 535 nm (emission). Trolox was used as the reference standard. The net area under the curve of decay of fluorescein was determined using the calibration curve of trolox (y = 0.035× + 0.08, R^2^ = 0.999) and expressed as mg trolox equivalents (TE)/g of extract.

#### Determination of Total polyphenolic content

The total polyphenolic content (TPC) of plant extracts was quantified by the modified Folin-Ciocalteu method [26]. Extracts were assayed within the assay concentration range of 50–500 μg/mL. Plant extracts (110 μL) were incubated with folin-ciocalteu reagent and sodium carbonate solution (10% *w*/*v*, 70 μL) for 30 min at room temperature (25 ± 2 °C). Absorbance was recorded at 765 nm. TPC was calculated using the calibration curve of Gallic acid standard curve (y = 0.053× + 0.105, R^2^ = 0.993) and expressed as mg gallic acid equivalents (GAE)/g of extract.

#### Determination of Total flavonoid content

The total flavonoid content (TFC) of plant extracts was quantified by the aluminium chloride method [27]. Extracts were tested within the assay concentration range of 50–500 μg/mL. Extracts (100 μL) were incubated with AlCl_3_-methanol solution (2%, 100 μL) for 10 min at room temperature (25 ± 2 °C) and absorbance was recorded at 415 nm. TFC was calculated using a calibration curve of quercetin (y = 0.033×–0.002, R^2^ = 0.999) and expressed in terms of mg quercetin equivalents (QE)/g of extract.

#### Gas chromatography - mass spectroscopy (GC-MS) analysis

The total ethanol extract of bark of *F. indica* was analysed by GC-MS using Thermoscientific Trace 1300 GC system, coupled with ISQ QD mass detector (EI mode, mass range of m/z 40–450). The GC system is equipped with a programmable temperature vaporization (PTV) inlet and a Supelcowax capillary column (30 m × 0.25 mm × 0.25 μm), fused with silica and polyethylene glycol as the stationary phase.

Sample was dissolved in ethanol (0.60 g/mL) and 500 μL of head space gas was introduced to the PTV inlet. The injector temperature was set at 250 °C with an initial oven temperature of 60 °C, which was set to increase at a rate of 5 °C min^− 1^ to reach up to 220 °C. Helium was used as the carrier gas (Flow rate: 1 mL min^− 1^). The compounds in the extract were matched and identified using mass spectral database of NIST 11, USA.

#### Analysis of phenolic compounds using high Performanace liquid chromatography (HPLC)

The ethanol extract of *F. indica* was dissolved in methanol (5 mg/mL) and filtered through a membrane filter (0.25 μm) for HPLC analysis. The HPLC system was equipped with a Agilent 1260 Infinity II system, consisting of a quaternary pump (G7111A), vial sampler, coloumn heater and Diode array detector (WR G7115A). Separation was achieved on a reversed phase coloumn C 18 (250 mm × 4.6 mm × 5 μm). The eluates were detected at 254, 280 and 320 nm. Two solvent mixtures were used as the mobile phase in a gradient system. Water/formic acid (1000/0.005 *v*/v) was used as solvent A and methanol was used as solvent B. The total flow rate was 0.5 mL/min. The gradient profile of the mobile phase was from 10% B linearly to 70% B in 60 min followed by an isocratic flow for 10 min and back to 10% B at 90 min followed by isocratic flow for 10 min to re-equilibrate [28].

#### Statistical analysis

All analysis was carried out in triplicate and experimental results were expressed as mean ± standard error (SE), analysed with one-way ANOVA. Turkey’s multiple range tests was applied for mean separation, when ANOVA was significant (*p* < 0.05). IC_50_ values were calculated using linear regression analysis. Pearson’s correlation coefficient was used for the correlation analysis (*p* < 0.05) (IBM SPSS Statistics 22.0).

## Results

### Enzyme inhibitory activities of selected medicinal plants

#### Arachidonate 5-lipoxygenase inhibitory activity

**Fig. 1:**
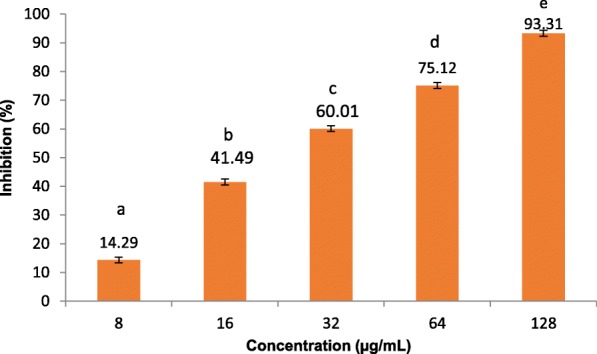
A5-LOX inhibitory activities of ethanol extract of bark of *Flacourtia indica.* Results are presented as mean ± SE (*N* = 3). Means followed by the same letter are not significantly different at *p* < 0.05

Based on the percent inhibition in the screening, the results revealed that, the ethanol bark extract of *F. indica* had the highest A5-LOX inhibitory activity followed by the extracts of *S. cochinchinesis* and *C. innophyllum*, while the extract of *T. cordifolia* had the lowest activity (Table 2). Apart from the extracts of *A. pedunculata, L. zeylanica* and *B. nodiflorus*, the other extracts showed high to moderate A5-LOX inhibitory activities. Based on the IC_50_ values, the extract of *F. indica* (Fig. 1) showed the highest A5-LOX inhibitory activity followed by *S. cochinchinesis* and *C. innophyllum* in a dose dependent manner confirming the results of the initial screening (Table 2). The activities of the extracts were found to be significantly different from the positive control baicalein, which showed a strong dose dependent activity against A5-LOX enzyme activity.

**Table 2:** Anti-A5-LOX activity of ethanol extracts of medicinal plants

Plant name	A5-LOX Inhibiton (%)^*^	Anti-A5-LOX activity IC_50_ (μg mL^− 1^)
*Sphaeranthus indicus*	32.45 ± 0.45^a^	136.69 ± 1.79^a^
*Acronychiapedunculata*	17.45 ± 0.94^b^	294.68 ± 2.23^b^
*Calophyllum innophyllum*	67.14 ± 0.51^c^	74.82 ± 1.35^c^
*Symplocos cochinchinesis*	70.12 ± 0.36^c^	39.01 ± 0.91^d^
*Tinospora cordifolia*	12.71 ± 0.71^b^	393.63 ± 1.74^e^
*Flacourtia indica*	89.35 ± 1.24^d^	22.75 ± 1.94^f^
*Leucus zeylanica*	19.37 ± 0.11^b^	258.03 ± 1.91^g^
*Barathranthus nodiflorus*	9.38 ± 0.32^e^	213.27 ± 1.55^h^
*Diospyros ebenum*	34.91 ± 0.84^a^	143.76 ± 1.03^a^
*Argyreia populifolia*	32.89 ± 0.17^a^	152.41 ± 1.00^i^
Baicalein	97.64 ± 0.65^f^	1.76 ± 0.15^j^

Data represented as mean ± SE (*N* = 3). *A5-LOX Inhibition at 100 μg/mL. Mean within each column followed by the same letter are not significantly different at *p* < 0.05

#### Hyaluronidase inhibitory activity

The hyaluronidase inhibitory activities ranged from 16.27 ± 1.00 to 42.31 ± 2.00% for the tested plant extracts. The ethanol bark extracts of *B. nodiflorus* and *D. ebenum* showed the highest activities followed by the extracts of *A. pedunculata* and *F. indica* (Table 3). The extracts of *S. indicus*, *C. innophyllum* and *T. cordifolia* showed no inhibitory activities against hyaluronidase enzyme (Table 3). The extracts of *B. nodiflorus* and *D. ebenum* showed moderate activities (Table 3) compared to the reference standard tannic acid and good, comparable activities when compared with the reported activity of indomethacine (50%, 500 μg/mL) [28] a clinical drug in use against inflammation.

**Table 3:** Xanthine oxidase and hyaluronidase enzyme inhibitory activities of total ethanol extracts of medicinal plants

Plant Name	Xanthine oxidase inhibition	Hyaluronidase inhibition
*Sphaeranthus indicus*	30.35 ± 0.32^a,f^	NI
*Acronychia pedunculata*	7.86 ± 0.14^b,d^	36.60 ± 1.02^a^
*Calophyllum innophyllum*	38.95 ± 1.28^c^	NI
*Symplocos cochinchinesis*	44.86 ± 1.43^c^	27.49 ± 1.09^b^
*Tinospora cordifolia*	17.92 ± 1.73^d^	NI
*Flacourtia indica*	70.46 ± 0.18^e^	36.67 ± 2.23^a^
*Leucus zeylanica*	13.26 ± 0.25^d^	24.38 ± 2.09^b^
*Barathranthus nodiflorus*	6.26 ± 0.93^b^	42.31 ± 2.00^d^
*Diospyros ebenum*	24.76 ± 2.16^a^	41.60 ± 1.18^d^
*Argyreia populifolia*	32.79 ± 2.16^f^	16.27 ± 1.00^e^
Allopurinol	99.26 ± 0.72^g^	NA
Tannic acid	NA	90.69 ± 0.50^f^

Inhibition (%) of xanthine oxidase and hyaluronidase is recorded at 250 and 500 μg/mL assay concentrations respectively

Data represented as mean ± SE (*N* = 3). Means followed by the same letter are not significantly different at *p* < 0.05, *NA* Not applicable

#### Xanthine oxidase inhibitory activity

**Fig. 2:**
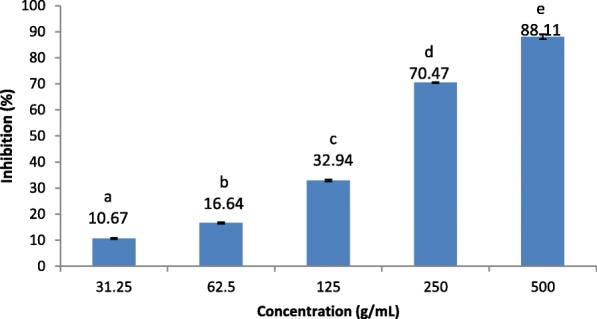
Xanthine oxidase inhibitory activities of ethanol extract of bark of *Flacourtia indica.* Results are presented as mean ± SE (*N* = 3). Means followed by the same letter are not significantly different at *p* < 0.05

All extracts showed some inhibitory activity against xanthine oxidase enzyme and activities ranged from 6.26 ± 0.93 to 70.74 ± 0.95%. The results revealed that, the ethanol bark extract of *F. indica* had the highest inhibitory activity followed by the extracts of *S. cochinchinesis* and *C. innophyllum* (Table 3). The extracts of *A. pedunculata* and *A. populifolia* showed significantly low inhibitory activities against xanthine oxidase enzyme with respect to the reference standard allopurinol (Table 3). The extract of *F. indica,* which showed the highest xanthine oxidase inhibitory activity showed significant (*p* < 0.05), dose dependent inhibitions within the concentration range of 31.25–500 μg/mL with a IC_50_ value of 176.62 ± 0.7 μg/mL (Fig. 2; Allopurinol: IC_50_: 2.33 ± 0.51 μg/mL)_*.*_

#### Nitric oxide production inhibitory activity and viability of LPS-activated RAW 264.7 macrophages

The inhibitory activities of extracts were moderate to low in comparison to the reference standard L-NMMA and ranged from 3.63 ± 0.69% to 38.07 ± 0.93%. Of the tested plant extracts, the extracts of *F. indica*, *S. cochinchinesis* and *C. innophyllum* showed significantly high activities, while that of *S. indicus, T. cordifolia*, *L. zeylanica* and *B. nodiflorus* showed significantly low activities (Table 4). The extracts showed no cytotoxicity (Cell viability: > 80%) at the tested concentration (Table 4).

**Table 4:** Inhibitory activities of ethanol extracts of medicinal plants on nitric oxide production and viability of LPS-activated RAW264.7 macrophages

Plant name	Nitric oxide production (μM)	% NO inhibition	% Cell viability
*Sphaeranthus indicus*	31.11 ± 0.17	9.46 ± 0.21^a,b^	87.21 ± 0.50
*Acronychia pedunculata*	30.39 ± 0.07	11.56 ± 0.21^b^	89.76 ± 0.35
*Calophyllum innophyllum*	23.67 ± 0.27	31.12 ± 0.78^c^	88.89 ± 0.82
*Symplocos cochinchinesis*	22.50 ± 0.28	34.52 ± 0.78^c^	82.15 ± 0.31
*Tinospora cordifolia*	32.89 ± 0.24	2.40 ± 0.69^a^	94.56 ± 2.57
*Flacourtia indica*	21.28 ± 0.32	38.07 ± 0.93^c^	87.31 ± 0.50
*Leucus zeylanica*	33.11 ± 0.24	3.63 ± 0.69^a^	93.02 ± 0.67
*Barathranthus nodiflorus*	31.22 ± 0.25	9.13 ± 0.72^a,b^	87.11 ± 1.16
*Diospyros ebenum*	29.08 ± 0.29	15.36 ± 0.85^d^	85.43 ± 1.22
*Argyreia populifolia*	27.64 ± 0.15	19.56 ± 0.43^d^	86.10 ± 0.61
L-NMMA	0.97 ± 0.12	97.10 ± 0.56^e^	97.54 ± 0.47

Data represented as mean ± SE (*N* = 3). Mean within each column followed by the same letter are not significantly different at *p* < 0.05. *Inhibition (%) at the assay concentration of 500 μg/mL

#### Oxidative burst inhibition

**Fig. 3:**
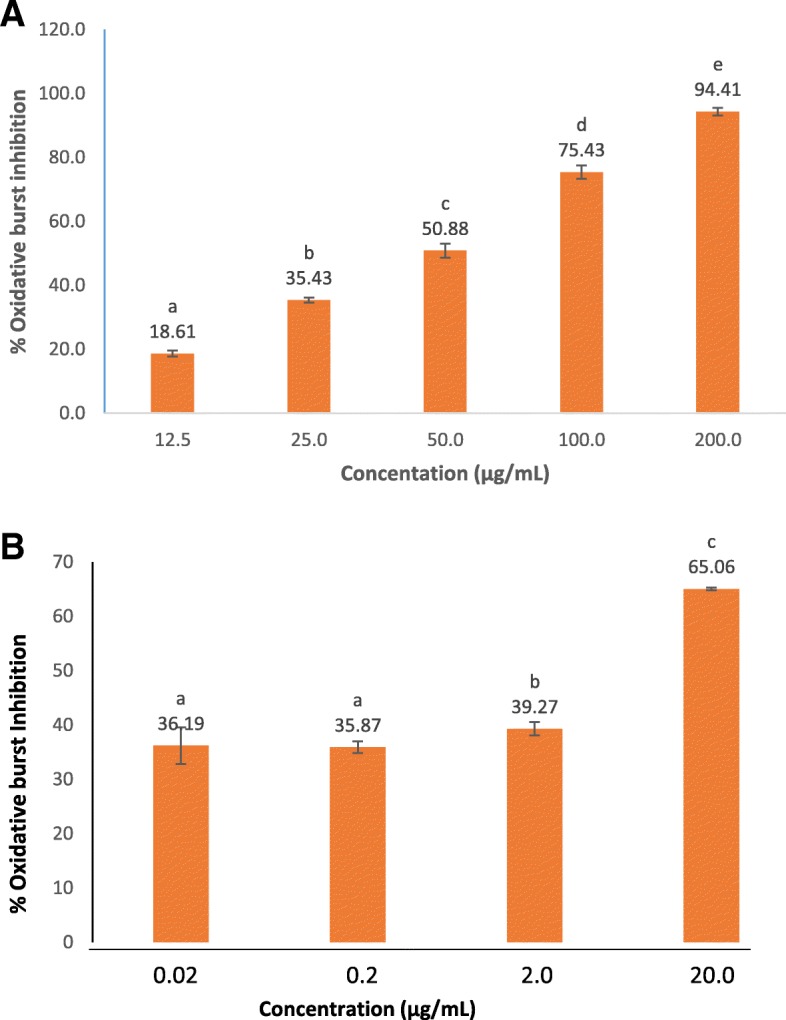
Oxidative burst inhibitory activities of ethanol extract of bark of *Flacourtia indica* on human whole blood (**a**) and polymorphoneutrophils (**b**). Results are presented as mean ± SE(N = 3). Means followed by the same letter are not significantly different at *p* < 0.05

The ethanol bark extract of *F indica*, which was identified as a highly anti-inflammatory extract was assessed for the effect on oxidative burst response on human whole blood and isolated PMNs. The extract showed a significant inhibition of ROS production on human whole blood (IC_50_: 47.64 ± 2.32 μg/mL), which was found to be dose-dependent (12.5–200 μg/mL) (Fig. 3a). Interestingly, the extract showed promising, significant oxidative burst inhibitory effect when tested on isolated PMNs (IC_50_:5.02 ± 0.38 μg/mL) (Fig. 3b), which is comparable with the reference drug Ibuprofen (IC_50_: 5.12 ± 0.45 μg/mL).

#### Anti-oxidant activity

Anti-oxidant capacities of the extracts were evaluated using four different methods including the DPPH free radical scavenging, FIC, FRAP and ORAC assays. The extracts showed high to low DPPH free radical scavenging activities, having the IC_50_ values within the range of 6.99 ± 0.02–743.49 ± 1.94 μg/mL. The highest DPPH free radical scavenging activities were showed by the extracts of *C. innophyllum* and *S. cochinchinesis* followed by the extracts of *F. indica* and *S. indicus* in comparison to the reference standard trolox (Table 5).

**Table 5:** Antioxidant activities of ethanol extracts of medicinal plants

Plant Name/Standard	IC _50_ (μgmL^− 1^)/Inhibition (%)^*^		FRAP values (mg TE/g)	ORAC values (mg TE/g)
	DPPH	FIC		
*Sphaeranthusindicus*	109.33 ± 1.19^a^	19.21 ± 0.56*	326.15 ± 3.53^a^	1018.71 ± 9.96^a^
*Acronychiapedunculata*	743.49 ± 1.94^b^	974.56 ± 2.31^a^	741.64 ± 1.75^b^	322.67 ± 1.94^b^
*Calophyllum nnophyllum*	6.99 ± 0.02^c^	19.50 ± 0.71*	2613.00 ± 7.23^c^	2111.0 ± 6.35^c^
*Symplocos cochinchinesis*	9.85 ± 0.28^c^	1093.53 ± 4.04^b^	2181.61 ± 2.16^d^	2910.7 ± 12.9^d^
*Tinospora cordifolia*	389.20 ± 0.75^d^	NI	586.66 ± 3.29^e^	121.29 ± 2.12^e^
*Flacourtia indica*	26.37 ± 0.49^e^	8.29 ± 0.26*	375.20 ± 2.79^f^	1480.20 ± 11.5 ^f^
*Leucus zeylanica*	352.65 ± 2.12^f^	33.46 ± 0.66*	157.69 ± 1.85^g^	63.69 ± 1.16^g^
*Barathranthus nodiflorus*	282.22 ± 1.78^g^	33.37 ± 0.40 *	427.29 ± 2.07^h^	18.07 ± 0.42^h^
*Diospyros ebenum*	177.32 ± 1.03^h^	NI	369.18 ± 0.61^f^	95.24 ± 0.00^e,g^
*Argyreia populifolia*	288.81 ± 1.45^g^	NI	268.01 ± 1.53^i^	479.55 ± 1.80 ^i^
Trolox	5.29 ± 0.09^c^	NA	NA	NA
EDTA-Na_2_	NA	13.07 ± 0.64 ^c^	NA	NA
GreenTea	NA	NA	NA	1662.82 ± 0.22^j^

Data represented as mean ± SE (*N* = 3). Mean within each column followed by the same letter are not significantly different at *p* < 0.05. *Inhibition (%) at the assay concentration of 1000 μg/mL, *NA* Not applicable

In FIC assay, the extracts showed low chelating activities, indicating high IC_50_ values in comparison to the reference standard EDTA-Na_2_. The extracts of *T. cordifolia, D. ebenum* and *A. populifolia* showed no chelating activity within the tested assay concentration range of 100–5000 μg/mL. Dose dependent activities were showed by the extracts of *A. pedunculata* and *S. cochinchinesis*. For the remaining extracts, which showed chelating activity, dose response studies were not carried out due to the interference of turbidity of the reaction mixture at higher concentrations (> 1000 μg/mL) (Table 5).

In FRAP assay, the extracts showed low to high reducing power within the range of 157.69 ± 1.85–2613.00 ± 7.23 mg TE/g. The extract of *C. innophyllum* showed the highest FRAP value followed by the extracts of *S. cochinchinesis* and *A. pedunculata*. The extract of *L. zeylanica* showed the lowest FRAP (Table 5).

The ORAC of extracts ranged from 18.07 ± 0.42–2910.7 ± 12.9 mg TE/g. The extract of *S. cochinchinesis* showed the highest among the extracts and even significantly higher ORAC in comparison to the standard green tea extract. The extracts of *C. innophyllum, F. indica* and *S. indicus* also showed high ORAC values and that of *L. zeylanica* showed the lowest ORAC, compared to other extracts (Table 5).

#### Total polyphenol and flavonoid contents

**Fig. 4:**
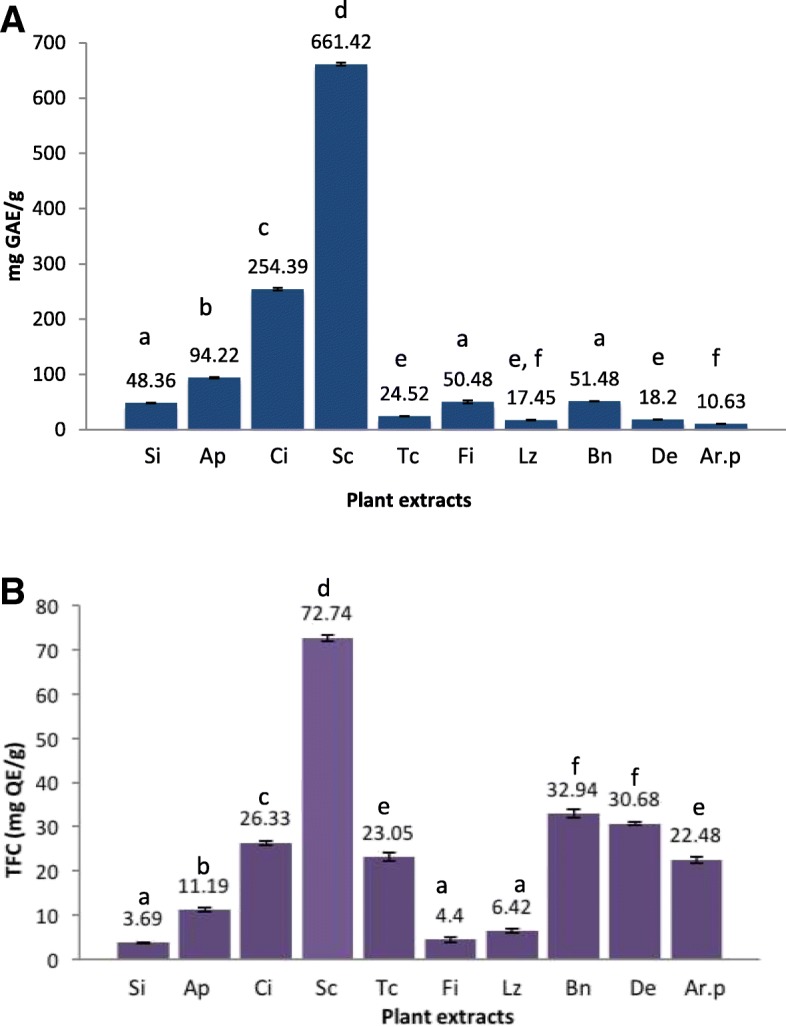
Total polyphenolic contents (**a**) and total flavonoid Contents (**b**) of plant extracts. Results are presented as mean ± SE (*N* = 3). Means followed by the same letter are not significantly different at *p* < 0.05. Si*:Sphaeranthus indicus,* Ap*:Acronychia pedunculata,* Ci*: Calophyllum innophyllum,* Sc*: Symplocos cochinchinesis,* Tc*:Tinospora cordifolia,* Fi*: Flacourtia indica,* Lz*: Leucus zeylanica,* Bn*:Barathranthus nodiflorus,* De*: Diospyros ebenum, Ar.p: Argyria populiflia.*

The total polyphenol contents of the extracts ranged from 10.63 ± 0.22–661.42 ± 2.67 mg GAE/g. The extract of *S. cochinchinesis* showed the significantly highest polyphenol content followed by that of *C. innoplyllum* and *A. pedunculata*. The extracts of *L. zeylanicus* and *D. ebenum* showed the lowest polyphenolic contents (Fig. 4).

The flavonoid contents of the extracts ranged from 3.69 ± 0.15–72.74 ± 0.76 mg QE/g. The extract of *S. cochinchinesis* showed the highest flavonoid content and that of *S. indicus* and *F. indica* showed the lowest flavonoid contents. The extracts of *B. nodiflorus* (32.94 ± 0.88 mg QE/g) and *D. ebenum* (30.68 ± 0.30 mg QE/g) also showed significant flavonoid contents (Fig. 4).

#### Correlation between assays

The correlation analysis is important to get an understanding of statistical relationships between different assays. The *p* values resulted from the correlation analysis among ten assays are given in Table 6.

**Table 6:** Pearson’s correlation coefficients of in-vitro anti-inflammatory activities, antioxidant activities, total phenolic and total flavonoids content of extracts

	LOX	HYL	XO	NO	DPPH	FRAP	FIC	ORAC	TPC	TFC
A5-LOX	1	0.327^ns^	0.930^**^	0.950^**^	−0.736^**^	.545^**^	.150^ns^	.844^**^	.540^**^	0.228^ns^
HYL		1	0.326^ns^	0.371^*^	−0.022^ns^	-.297^ns^	.115^ns^	-.043^ns^	-.081^ns^	−0.059^ns^
XO			1	0.864^**^	−.728^**^	.287^ns^	-.041^ns^	.696^**^	.326^ns^	0.069^ns^
NO				1	−.656^**^	.592^**^	.243^ns^	.829^**^	.587^**^	0.368^*^
DPPH					1	−.417^*^	.270^ns^	−.675^**^	−.416^*^	−0.324^ns^
FRAP						1	.429^*^	.811^**^	.810^**^	0.607^**^
FIC							1	.436^*^	.707^**^	0.520^**^
ORAC								1	.857^**^	0.523^**^
TPC									1	0.821^**^
TFC									.	1

The statistical significance is represented with ^**^p < 0.01 and ^*^p < 0.05. *ns* non-significant

#### Gas chromatography - mass spectroscopy (GC-MS) analysis of ethanol extract of bark of *Flacoutia indica*

**Fig. 5:**
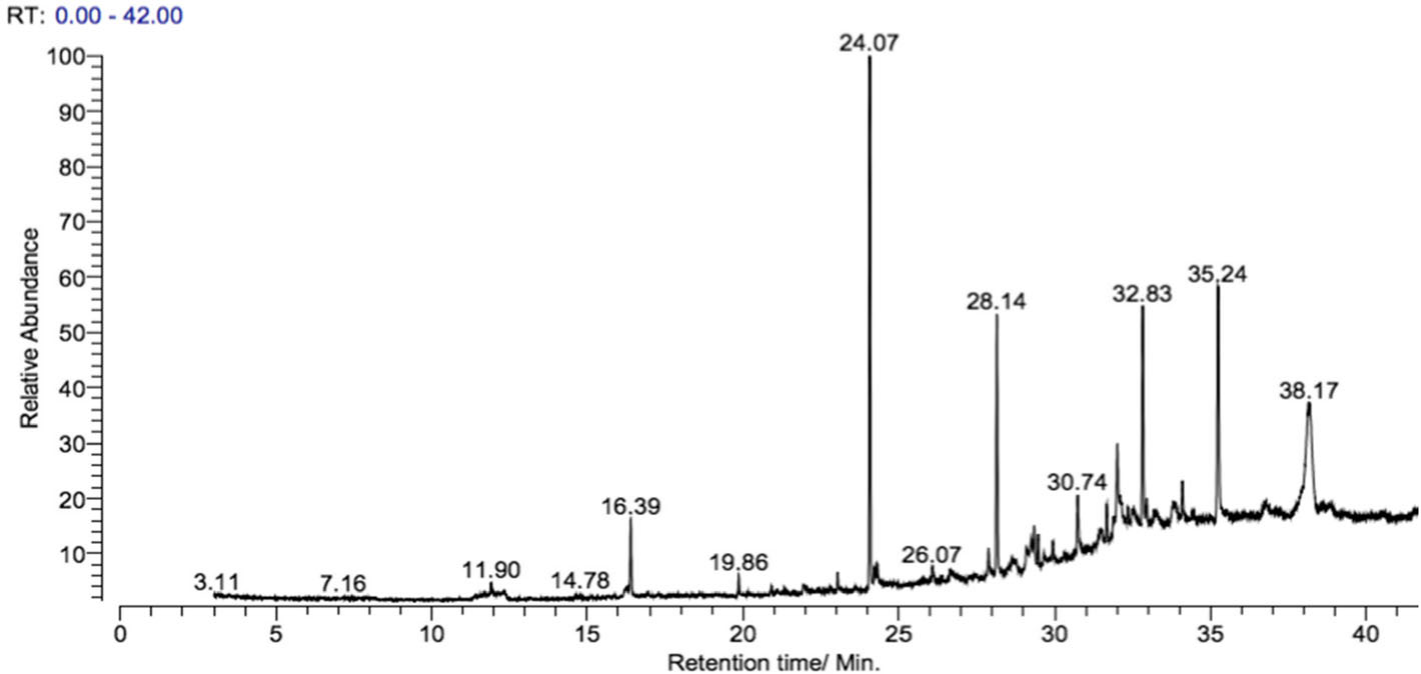
Gas Chromatography - Mass Spectroscopy (GC-MS) spectrum of ethanol bark extract of *Flacourtia indica*

The GC-MS analysis of ethanol extract of bark of *F. indica* revealed the presence of six phytoconstituents including acid esters and fatty acid derrivatives (Table 7). Propan-2-yl tetradecanoate, [1,1’-Bicyclopropyl]-2-octanoic acid, 2′-hexyl-, methyl ester, Linoleic acid ethyl ester, Hexadecanoic acid, ethyl ester and Benzoic acid, ethyl ester were identified as major compounds (> 2% peak area) (Fig. 5).

**Table 7:** GC-MS data of ethanol bark extract of *Flacutia indica*

Retention time	Name of the compound	Molecular formula	Peak area %
16. 39	Benzoic acid, ethyl ester	C_9_H_10_O_2_	2.42
24. 07	Propan-2-yl tetradecanoate	C_17_H_34_O_2_	16.27
27. 87	Estra-1,3,5(10)-trien-17á-ol	C_18_H_24_O	0.94
28. 14	Hexadecanoic acid, ethyl ester	C_18_H_36_O_2_	8.29
32. 83	Linoleic acid ethyl ester	C_20_H_36_O_2_	7.72
38. 17	[1,1’-Bicyclopropyl]-2-octanoic acid, 2′-hexyl-, methyl ester	C_21_H_38_O_2_	12.56

#### Analysis of phenolic compounds using high performance liquid chromatography (HPLC)

**Fig. 6:**
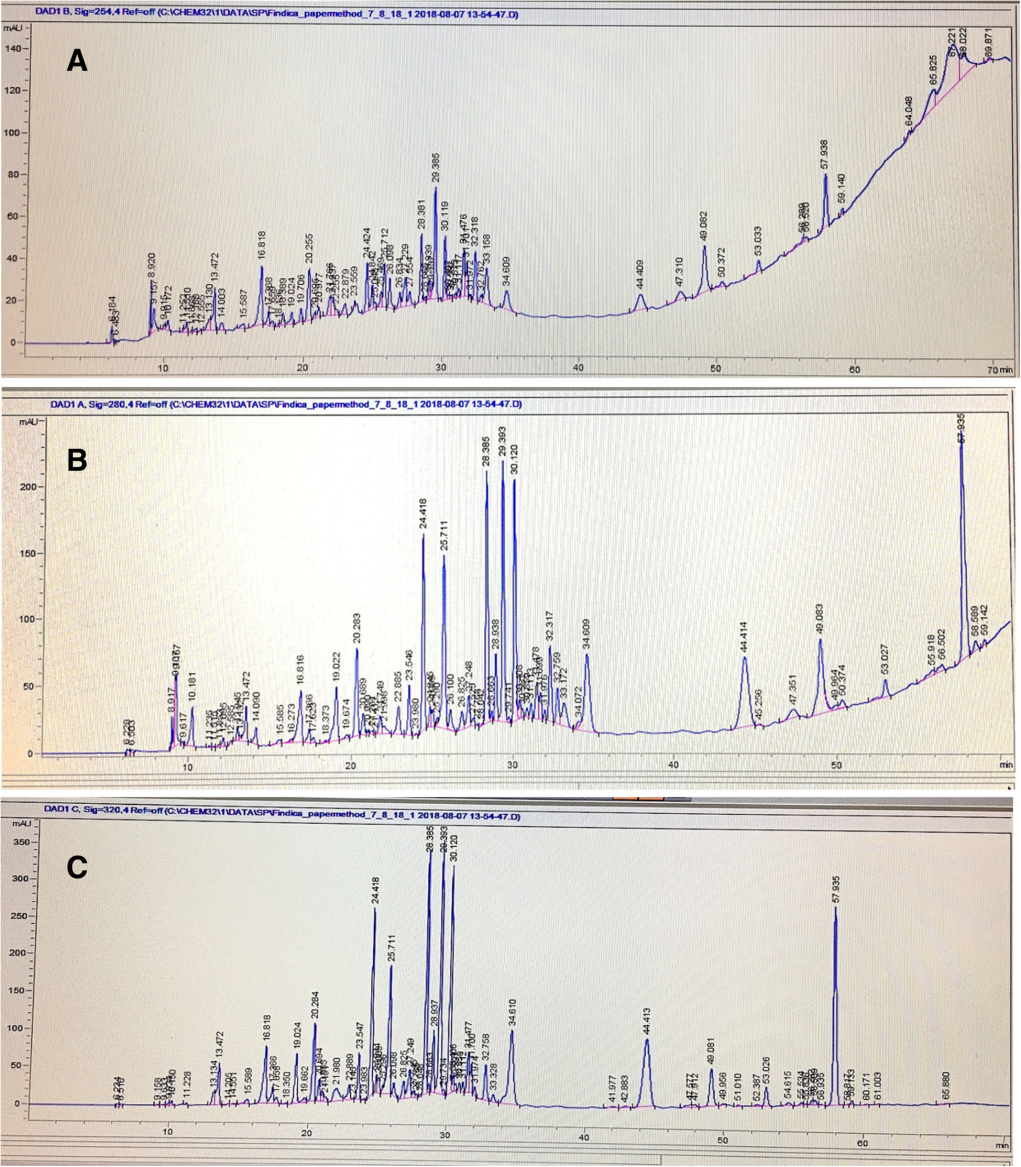
HPLC chromatogram of ethanol bark extract of *Flacourtia indica*

The HPLC analysis produced a well resolved chromatogram representing peaks corresponding to retention time of phenolic compounds. The HPLC chromatogram of ethanol bark extract of *F. indica* at 254, 280 and 320 nm is given in Fig. 6.

## Discussion

### Enzyme inhibitory activities of selected medicinal plants

#### Arachidonate 5-lipoxygenase inhibitory activity

In this assay, all the extracts showed significant (*p* < 0.05) A5-LOX inhibitory activities at the tested concentrations. The ethanol bark extract of *F. indica* showed the highest A5-LOX inhibitory activity followed by that of *S. cochinchinesis* over the other extracts. When compared with the reported A5-LOX inhibitory activity of Caffeic acid, (IC_50_: 57.0 μg/mL), [29] which is a known lipoxygenase inhibitor, the IC_50_ values of bark extracts of *F. indica* and *S. cochinchinesis* are about 2.5 fold and 1.5 fold lower than that of caffeic acid, respectively so that could be considered as even more potent than caffeic acid as A5-LOX inhibitors. To best of our knowledge, no previous studies have been conducted on A5-LOX inhibitory potential of extracts of *F. indica* and *S. cochinchinesis.*

#### Hyaluronidase inhibitory activity

In the hyaluronidase inhibitory assay, the extracts showed moderate to low anti-hyaluronidase activities at the tested concentration in comparison to the reference standard tannic acid. The extracts of *B. nodiflorus* and *D. ebenum* exhibited the highest anti-hyaluronidase activities compared to the other extracts. *B. nodiflorus* and *D. ebenum* are two medicinal plants endemic to Sri Lanka, that have been less exploited in the field of scientific research. The hyaluronidase inhibitory properties of these two plant extracts are recorded for the first time to upgrade the medicinal value of the species.

#### Xanthine oxidase inhibitory activity

All the extracts studied showed significant xanthine oxidase inhibitory activity (p < 0.05). The extract of *F. indica* showed the highest, xanthine oxidase inhibitory activity compared to the other extracts tested. This promising anti-xanthine oxidase potential of the extract of *F. indica* may be further supported by the traditional use of extracts of *F. indica* in the treatment of gouty arthritis. Specifically bark extracts have been used in the treatment of gout in the Unani system of medicine, where xanthine oxidase enzyme plays a major role in pathogenesis by imparting inflammation and catalyzing the formation of uric acid crystals leading to arthritic conditions [30].

#### Nitric oxide production inhibitory activity and viability of LPS-activated RAW 264.7 macrophages

Of the studied extracts, *F. indica* bark showed the highest NO production inhibitory activity. The NO production inhibitory activity could be either because of the direct inhibition of iNOs enzyme catalytic activity or expression of nitric oxide synthase. The high cell viability observed in the MTT cytotoxicity assay is evident for the non-toxic nature of the tested extracts confirming, that the observed NO production inhibitions are not due to any cytotoxic effect of extracts.

#### Oxidative burst inhibitory activity

The bark extract of *F. indicia,* showed a pronounced oxidative burst inhibition in comparison to ibuprofen, when tested on PMNs. This observed activity may be due to the inhibition of NADPH oxidize enzyme, which catalyzes the generation of ROS or the direct scavenging of ROS upon the stimulation of opsonized zymosan [31].

#### Anti-oxidant activity

The bark extract of *C. innophyllum* showed the highest DPPH free radical scavenging activity and FRAP over other extracts, which may be attributed to the presence of major chemical compounds such as xanthones and coumarins abundantly present in the extracts [23]. *C. innophyllum* is known as a medicinal plant with number of curative properties and it has been extensively studied worldwide [23]. The present study ascertains the significant free radical scavenging activity of the Sri Lankan variety of *C. innophyllum* free radical scavenging activity with the means of marked DPPH free radical scavenging and FRAP. Lower chelating properties of the extracts may be due to the low contents of effective metal chelating compounds in the extracts.

ORAC evaluates a hydrogen atom transfer mechanism of anti-oxidants [25]. Significant ORAC of extracts indicates the peroxyl radical absorbance capacities of the extracts at different degrees. The extract of *S. cochinchinesis* showed the highest ORAC over the other extracts as well as the standard green tea extract. The presence of triterpenoids and flavonoid glycosides may be attributed to the anti-oxidant potential of the extracts of *S. cochinchinesis.* It has been extensively studied for its anti-diabetic properties [32].

#### Total polyphenol and flavonoid contents

The moderate to weak and non-significant correlations of antioxidant and enzyme inhibitory activities with TPC and TFC (Table 6), respectively suggested that the polyphenolic and flavonoid compounds of the extracts may not be solely responsible for the enzyme inhibitory and antioxidant activities. It is further confirmed by the fact, that the ethanol extract of bark of *F. indica* has exhibited the highest bio-activities irrespective of possessing moderate to low contents of polyphenols and flavonoids (Fig. 4) Among the identified phytoconstitutents, hexadeconoic acid, ethyl ester is known to possess anti-oxidant properties as well as other bio-activities such as hypocholesterolemic, nematicide, pesticide, anti-androgenic, hemolytic and 5-alpha reductase inhibitory properties. Also the compound has flavor properties [33] which may be attributed to the strong aroma of the ethanol bark extract of *F. indica*. Linoleic acid ethylester is another bio-active compound detected in the extract of bark of *F. indica* which is known to possess anti-inflammatory properties and many more of bio-activities including anti-arthritic, anti-histaminic, anti-eczemic and anti-acne properties [33].

#### Correlation between assays

The A5-LOX, XO and NO inhibitory activities of plant extracts showed high, negative and significant correlations with DPPH free radical scavenging activity (IC_50_ values) (*r* = − 0.736, − 0.728 and − 0.656 respectively at *p* < 0.01) and high, positive and significant correlations with ORAC assay (*r* = 0.844, 0.796 and 0.696 respectively at *p* < 0.01). This observation may be indicative of the dual function of bio-actives of plant extracts as free radical, peroxyl radical scavenging and A5-LOX, XO and NO production inhibitory compounds. Moreover, the A5-LOX, XO and NO inhibitory activities of plant extracts have showed high, positive and significant correlations with each other supporting the fact, that some common group of bio-actives of the extracts could be attributed for the enzyme inhibitory activities of the extracts (Table 6). FRAP of the extracts has showed a high, positive and significant correlation with TPC (*r* = 0.810, *p* < 0.01) so that the FRAP of the extracts may be due to the presence of polyphenols, which are well known to participate in redox reactions.

#### Gas chromatography - mass spectroscopy (GC-MS) analysis of ethanol extract of bark of *Flacoutia indica*

In previous studies the GC-MS analysis of methanol extract of root of *F. indica* has showed the presence of 4-Benzyol-3-methoxyisocoumarin as the major compound [34], which was not detected in the bark extract in this study. No other GC-MS analysis has been previously carried out on any species of the genus *Flacourtia* except for root of *F. indica* and this is the first report of the GC-MS analysis of Bark of *F. indica*.

#### Analysis of phenolic compounds using high performance liquid chromatography (HPLC)

According to a reported study, liquid chromatography-mass spectroscopy (LC-MS^n^) analysis of aqueous methanolic fruit extract of *F. indica* has enabled identification of 35 phenolic compounds including rutin, feruloylquinic acid, esculin, gentisic acid glycoside, salicylic acid glycoside and derivatives of caffeolyquinic acids, quercetin and kaempferol [28]. The HPLC peak pattern of the ethanol bark extract of *F. indica* showed similarities with the reported HPLC peak pattern of aqueous methanolic fruit extract of *F. indica* based on the retention times of the peaks under similar experimental conditions. Therefore, the reported compounds of the fruit extract may also be present in the bark extract of *F. indica.* Further, the HPLC chromatogram may be used for standardization of ethanol bark extract of *F. indica.* However, further chemical Characterisation is needed for the identification of the compounds and activity guided fractionation is in progress.

In addition, presence of several other bio-active phytoconstituents including phenolic glycosides [35] coumarins (scoparon) [36, 37] and different types of polyphenolic compounds having radical scavenging properties such as coumaroylglucopyranose, tannins and butyrolactones [38] in the extracts of *F. indica* have been reported. Phenolic glycosides and coumarins have long been recognized as potent A5-LOX and xanthine oxidase inhibitors as well as antioxidants [39]. The bark of *F. indica* has been traditionally used to treat rhumatoid arthritis, [40] which is meadiated by inflammation. According to previous studies, The extracts of *F. indica* are known to possess a broad range of pharmacological activities including anti-inflammatory properties [34–36, 41] yet the pharmacological profile needs to be further investigated using more in vivo, in vitro and clinical studies [40]. This is the first report investigating the bio-activities of extract from bark of *F. indica* except for one study in which, the methanol extract of bark of *F. indica* has shown good DPPH free radical scavenging activity (IC_50_: 17.5 ± 1.0 μg/ml) [38].

## Conclusion

The extracts showed significant anti-inflammatory activities in vitro in terms of A5-LOX, xanthine oxidase, hyaluronidase and nitric oxide inhibitory activities along with promising anti-oxidant activities. Among the ten extracts, the ethanol extract of bark of *F. indica* showed the highest anti-inflammatory activity with good radical scavenging activities. Therefore, the ethanol extract of bark of *F. indica* is identified as a source of anti-inflammatory agents, which will be further studied to isolate and characterize bio-active constituents.

To the best of our knowledge, through this study, the pro-inflammatory enzyme inhibitory and anti-oxidant potential of ethanol bark extract of *F. indica* was identified and analysed by GC-MS and HPLC for the first time and selected for further bio-logical and chemical characterization.

### Compounds studied

Benzoic acid, ethyl ester, Propan-2-yl tetradecanoate, Estra-1,3,5(10)-trien-17á-ol, Hexadecanoic acid, ethyl ester, Linoleic acid ethyl ester, [1,1’-Bicyclopropyl]-2-octanoic acid, 2′-hexyl-,methyl ester.

## Abbreviations

A5-LOX: Arachidonate-5-lipoxygenase
AAPH: Potassium persulphate,2,2-azobis (2-amidinopropane) dihydrochloride
DMEM: Dulbecco’s modified Eagle’s medium
DMSO: Dimethyl sulfoxide
DPPH: 2,2-diphenyl-2-picryl-hydrazyl
EDTA-Na_2_: Ethylenediamine tetra acetic acid disodium salt
FCS: Fetal calf serum
FIC: Ferrous ion chelating
FRAP: Ferric reducing antioxidant power
GC-MS: Gas chromatography-Mass spectrometry
HBSS: Hanks Balanced Salt Solution
HPLC: High performance liquid chromatography
LPS: Bacterial lipopolysaccharide,L-NMMA:NG-Monomethyl-L-argininep-dimethylamino benzaldehyde
MTT: 3-(4,5-dimethylthiazol-2-yl)-2,5-diphenyltetrazoliumbromide
NO: Nitric oxide
ORAC: Oxygen radical absorbance capacity
PDMAB: p-Dimethylaminobenzaldehyde
SOZ: Serum opsonized zymosan
XO: Xanthine oxidase
